# *Rumex dentatus* Inhibits Cell Proliferation, Arrests Cell Cycle, and Induces Apoptosis in MDA-MB-231 Cells through Suppression of the NF-κB Pathway

**DOI:** 10.3389/fphar.2017.00731

**Published:** 2017-10-12

**Authors:** Riffat Batool, Ejaz Aziz, Benny K.-H. Tan, Tariq Mahmood

**Affiliations:** ^1^Department of Plant Sciences, Faculty of Biological Sciences, Quaid-i-Azam University, Islamabad, Pakistan; ^2^Department of Pharmacology, Yong Loo Lin School of Medicine, National University of Singapore, Singapore, Singapore

**Keywords:** breast cancer, apoptosis, NF-κB, phosphorylation, *Rumex dentatus*

## Abstract

**Background:** *Rumex dentatus*, commonly known as tooth docked, is widely used in traditional system of medicines. Although it is well reported for its biological activities and medicinal value, only few studies have been carried out to assess its anticancer potential.

**Purpose:** This study seeks to evaluate the anticancer activity of leaf extracts of *R. dentatus* against breast cancer MDA-MB-231 cell line, a triple negative human breast cancer cell line with invasive properties and to identify the molecular targets underlying its mechanism of action.

**Methods:** Cytotoxicity of plant extracts was determined against breast cancer cells, using the MTT assay. Flow cytometry was performed to analyze the changes in cell cycle and apoptotic effect, if any. Cells were also studied for their wound healing and invasive potential as well as for Western blotting of apoptotic genes and nuclear factor-kappaB (NF-κB) pathway.

**Results:** The results revealed that *R. dentatus* methanol (RM) and chloroform (RC) extracts of *R. dentatus* had the highest inhibition of cell proliferation in a concentration- and time-dependent manner. This inhibitory effect was found to be linked to arrest of cell cycle at the G0/G1 phase, along with induction of apoptosis and accumulation in the sub-G1 phase. Moreover, it was shown that both RM and RC inhibited the proliferation of the malignant cells and induced apoptosis by repressing the activation of NF-κB and its subsequent transcripts, Bcl-xl, Bcl-2, Cyclin D1, survivin, and XIAP. Apoptosis was also confirmed in the cells as suggested by caspase-3 detection. RM and RC also abrogated IκBa phosphorylation in the malignant cells as well as reduced the invasive and migratory capabilities of these cells.

**Conclusion:** Our findings suggest that the methanol and chloroform extracts of *R. dentatus* may have anti-cancer compounds that are potentially useful in the treatment of human breast cancer.

## Introduction

Despite significant progress in modern cancer research, breast cancer remains the most common malignancy and the major causative agent of death in women, especially in developed countries (Farshori et al., 2013). Although modern treatments have been found to be effective against breast cancer, these remedies are associated with adverse effects. The anthracycline, doxorubicin (DOX), which has high efficacy as an anti-breast cancer agent is associated with cardiotoxicity, including development of a cardiomyopathy (Weiss, 1992). This limits the chemotherapeutic use of DOX (Lefrak et al., 1973). In addition, most of the identified chemotherapeutic and hormone compounds are ineffective against cancer due to the occurrence of resistant malignant cells as well as their non-specific mechanisms of action (Lee and Azimahtol, 2003). It is thus necessary to identify new chemotherapeutic and/or chemopreventive alternatives that have minimal or no toxic effect on normal tissue and thereby have a more favorable therapeutic window.

In this regard, plant extracts have been used as an effective source for the identification of anti-cancer compounds. Lee and Azimahtol (2003) reported that styrylpyrone derivatives isolated from *Goniothalamus* sp. showed significant anti-proliferative activity against MCF-7 human breast cancer cell line. Many plant-derived natural products have been identified with anti-cancer properties that have been tested successfully against breast cancer in *in vitro* studies as well as in several epidemiological studies. In fact, multiple bioactive compounds that are derived from plants have been proven to be more beneficial than single pharmacological agents to combat breast cancer. Natural agents such as sulforaphane, resveratrol, and curcumin, among others, are gaining importance as adjuvant anti-cancer agents with minimal or no side effects (Vira et al., 2012).

The genus *Rumex* consists of more than 150 species of plants that are widely distributed among the globe. Some studies have reported the flavonoids and anthraquinones as major chemical constituents of this genus (Zhang et al., 2012). It includes many medicinally important plant species that are effective for the treatment of some dangerous diseases, including infection (Orhan et al., 2009). The antibacterial potency of *Rumex alpinus* and *Rumex caucasicus* was demonstrated against many food-borne bacterial diseases (Yildirim et al., 2001). *Rumex dentatus* of the family polygonaceae (commonly known as “toothed dock”) has allelopathic activity and produces some growth inhibitory substances that restrain the growth of adjacent plants. The shoots and leaves of *R. dentatus* and *R. hastatus* showed refrigerant properties (Hussain et al., 2006). The roots of *R. dentatus* have been widely used in skin disorders and as an astringent (Chopra et al., 1986). Moreover, various important bioactive compounds, such as chlorogenic acid, quercetin, myricitin, vitamin C, and kaempferol, have been identified in these roots. Saleem et al. (2014) demonstrated hepatoprotective effect of *R. dentatus* against paracetamol-induced liver damage in mice. Given the medicinal importance of *R. dentatus*, the purpose of the present study was to investigate it for possible anticancer activity, using the MDA-MB-231 breast cancer cell line.

## Materials and Methods

### Collection of Plant and Extract Preparation

Fresh specimen of the leaves of *R. dentatus* was collected from Khyber Pakhtunkhwa, Pakistan and a voucher specimen (HPBMBL-16-023) was stored in the Herbarium of Plant Biochemistry and Molecular Biology Laboratory, Quaid-i-Azam University, Islamabad. The specimen was thoroughly washed and dried under shade and then powdered with a grinder. Crude extracts of leaf part were prepared by soaking 25 mg of dried powder in ethanol, methanol, benzene, chloroform, and *n*-hexane (250 ml) for 1 week with irregular shaking. The extracted mixture was filtered through Whatman filter paper No. 1 and the residue was again soaked in the respective solvents for an additional 1 week. Process was repeated thrice; filtrates were combined and concentrated by evaporating the solvents under reduced pressure at 45°C in a rotary evaporator (Buchi, Switzerland). The five extracts of *R. dentatus* from *R. dentatus* methanol (RM), ethanol (RE), benzene (RB), chloroform (RC), and *n*-hexane (RH) were re-dissolved in 10% dimethyl sulfoxide (DMSO) before use.

### Reagents

Tris, glycine, sodium chloride (NaCl), sodium dodecyl sulfate (SDS), ammonium persulfate (APS), hepes, ethylenediaminetetraacetic acid (EDTA), albumin, mercaptoethanol-6, 100% triton-X, trypan blue, Dulbecco’s Modified Eagle’s Medium (DMEM), crystal violet, trypsin EDTA, Annexin V-FITC, 3-(4,5-dimethylthiazol-2-yl)-2,5-diphenyltertrazolium bromide (MTT), ribnuclease A (RNase A) from bovine pancreas, ABAM trypsin, ABAM, and propidium iodide (PI) were obtained from Sigma-Aldrich (St. Louis, MO, United States). Western blot membrane (0.45 mm), Bradford protein assay kit, TEMED, Laemmli sample buffer, and 40% acrylamide were acquired from Bio-Ras (Hercules, CA, United States). However, phosphate buffer saline (PBS) along with SDS was procured from Vivantis Technologies (Selangor, Malaysia). Likewise, fetal bovine serum (FBS) was obtained from Hyclone (Loughborough, United Kingdom) and Tween 20 from Merck & Co., Inc.

Besides these reagents, RNase and Cell Death Detection ELISA PLUS DNA fragmentation kit were supplied by Roche-Diagnostics (Mannheim, Germany) and TransAM nuclear factor-kappaB (NF-κB) p-65 along with nuclear extraction kit by Active Motif (Carlsbad, CA, United States). Chemiluminescent substrate for Western blotting was purchased from Advansta (Menlo Park, CA, United States). IL-6 ELISA kit was supplied by eBioscience (San Diego, CA, United States). Different antibodies such as survivin, Bcl-2, XIAP, Cyclin D1, Bcl-xL, chicken anti-mouse IgG horseradish peroxidase-linked antibody, IκBα chicken anti-rabbit IgG horseradish peroxidase-linked antibody, Akt, p-Akt (Thr308), and p-65 were supplied by Santa Cruz Biotechnology (Santa Cruz, CA, United States). Finally, β-actin, IKKα, IKKβ, pp-65 (Ser536), Tak, pTAK1, caspase-3, IκBα, p IκBα, cleaved caspase-3, and GAPDH were acquired from Cell Signaling Technology (Beverly, MA, United States).

### Cell Lines

The human breast cancer cell line MDA-MB-231 and a normal breast cell line MCF-10A were acquired from American Type Culture Collection (ATCC). These cells were grown in DMEM supplemented with 10% FBS along with 1% antimycotic (ABAM) and maintained under conditions of 37°C with 95% air and 5% CO_2_ in a tissue culture cabinet (Manassas, VA, United States).

### MTT Cell Proliferation Analysis Assay

To detect the inhibitory effect of *R. dentatus* on MDA-MB-231 cell line and MCF-10A (a normal breast cell line), MTT assay was carried out. The reactant cell lines were kept in 96-well plates for 24 h at a density of (5 × 10^4^) cells/well followed by treatment with 50, 100, 200, and 400 μg/ml of extracts for 24 and 48 h, respectively, along with negative control (0.5% of DMSO). Afterward, media were removed and 5 mg/ml MTT reagents in sterile PBS were loaded to all respective wells followed by 4 h incubation. Further, MTT solution was removed and DMSO was used to dissolve formazan precipitate. Finally, absorbance at 570 nm of each well was measured by scanning in a multi-well spectrophotometer (TECAN, Mannedorf, Switzerland).

### Cell Cycle Analysis

MDA-MB-231 and MCF-10A cells were seeded in duplicate at a density of 2 × 10^5^ cells/well and incubated for 24 h before drug treatment. After that, cells were exposed to the indicated drug concentrations for 12, 24, and 48 h to analyze cell cycle distribution. The treated cells were harvested by trypsinization followed by washing with PBS buffer and centrifugation at 4°C for 5 min at 200 × *g*. After overnight incubation at 4°C of fixed cells in chilled absolute ethanol, centrifugation was performed at 300 × *g* for 5 min and 1 ml of 1× PBS was used for re-suspension of pellet. 0.5 ml of RNase A was then applied to re-suspended cells for 20 min and subsequently stained with 1 mM PI at 37°C for 15 min. DNA content in a cell population was measured by flow cytometry (BD LSRFortessa^TM^ cell analyzer, United States) and cell cycle distribution was analyzed with Summit 4.3 software (Beckman Coulter, Inc.).

### Analysis of Apoptosis

The differentiation of necrotic and apoptotic cells was achieved via Annexin V-FITC kit (Miltenyi Biotec, Bergisch Gladbach, Germany). The reactant cells were kept in six-well plates for 24 h at a density of 2 × 10^5^ cells/well prior to treatment with *R. dentatus* extracts of different concentrations (50, 100, 200, and 400 μg/ml) for 12, 24, and 48 h, respectively. The cells were trypsinized, washed, and stained for 15 min with Annexin V-FITC and PI binding buffer. After that, washed stained cells were instantly visualized using flow cytometry (BD LSRFortessa^TM^ cell analyzer, United States). For each trial, 10,000 events per sample were detected.

### Caspase 3/7 Assay for Apoptosis Detection

Apoptosis was analyzed using caspase 3/7 assay kit (Promega Inc., United States) according to the user manual guidelines. MDA-MB-231 cells were incubated for 48 h with each selected plant extracts of *R. dentatus* along with control and incubated cells, and assessed for caspase 3/7 activity.

### DNA Fragmentation

DNA fragmentation in MDA-MB-231 cells was determined upon extract treatment to detect cellular apoptosis using Cell Death Detection ELISA Kit (Roche Diagnostic). For this, MDA-MB-231 cells were exposed with various concentrations of *R. dentatus* extracts for different time intervals followed by trypsinization and washing with PBS buffer. The treated cells were collected, suspended in lysis buffer (200 ml), and left to incubate at room temperature for 30 min followed by centrifugation at 5,000 × *g* for 10 min. The resultant supernatants (20 ml) were taken into streptavidin-coated 96-well microtiter plates along with immunoreagent mixture (80 ml). These plates were later placed in an incubator with continuous shaking (200 × *g*) at 25°C. In the next step, unbound antibodies were washed out and color was developed with ABTS substrate and absorbance was recorded at 405 nm. Further, percentage of apoptosis (enrichment factor) was calculated using the following formula:

Enrichment factor = DNA fragments in treated sample/DNA fragments in control cells.

### DNA Binding Assay

DNA binding assay was also performed with the help of TransAM NF-κB p-65 transcription factor assay kit (Active Motif, Carlsbad, CA, United States) as described in protocol. Briefly, 20 μg of nuclear extracts from *R. dentatus-*treated cells were pipetted into 96-well plates coated with oligonucleotide comprising NF-κB consensus-binding sequence (5′-GGGACTTTCC-3′) for were placed in incubator and kept at room temperature for 1 h. In subsequent step, plates were gently washed and incubated with NF-κB p-65 primary antibody for 1 h. Further for the detection of bound primary antibody, HRP-conjugated secondary antibody was added and spectrophotometer (Tecan Systems, San Jose, CA, United States) was used for quantification of sensitive colorimetric readout at 450 nm.

### Luciferase Assay

MDA-MB-231 cancer cells were loaded in 96-well plates at a density of 1 × 10^4^ cells per well in DMEM medium supplemented with 10% FBS for luciferase assay. The malignant cells were further transfected with dominant negative IκBa (IκBa-DN) as well as wild-type plasmid after 24 h of incubation. Luciferase activity of treated cells was performed with luciferase assay kit (Promega Inc., United States), visualized via Tecan plate reader (Durham, NC, United States) and normalized to β-galactosidase activity. All the results were obtained in triplicate.

### Western Blot Analysis

MDA-MB-231 cells were seeded in duplicate in six-well plates at 2 × 10^5^ cells per well followed by overnight incubation. After that *R. dentatus* extracts of specified concentrations were applied in malignant cells and incubated for 12, 24, and 48 h of time intervals. Protein from treated cancerous cells with different plant extracts was isolated using chilled lysis buffer (Beverly, MA, United States) supplemented with PMSF (protease inhibitor cocktail). To remove the insoluble residues from protein, the extracted lysates were centrifuged at 4°C for 5 min at 13,000 × *g*. The concentration of protein was measured with Bradford protein assay kit (Bio-rad, Hercules, CA, United States). Moreover, protein absorbance was also recorded at 595 nm with the help of microplate reader (TECAN infinite M200, Mannedorf, Switzerland) and estimation of protein quality was carried out based on bovine serum albumin (BSA) standard curve. Protein lysates were boiled for 5 min at 100°C along with Laemmli sample buffer (Bio-rad, Hercules, CA, United States) and resolution was done on 12% SDS polyacrylamide gels. Protein was then transferred via Electrophoretic Transfer Cell (Bio-rad, Hercules, CA, United States) to a nitrocellulose membrane at 15 V for 30 min. The membrane was blocked with blocking buffer by centrifugation (60 × *g*) for 30 min followed by washing of membrane at 90 × *g* for 5 min. Afterward, washed nitrocellulose membrane was probed with indicated primary antibodies (Cyclin D1, survivin, caspase-3, cleaved caspase-3, XIAP, TAK, pTAK1, IKKα, IKKβ, *IkBa*, P-*IkBa*, P-65, PP-65, Akt, and p-Akt) for protein of interest at 4°C for overnight. Consequently, blotted membrane was properly washed (three times) for 5 min at 90 × *g* prior to probing with anti-mouse/chicken anti-rabbit antibody (secondary antibodies) and diluted (1:1,000) with blocking buffer (Santa Cruz, CA, United States) at room temperature for 1 h followed by repetition of washing (thrice) at 90 × *g* for 5 min. Finally, the immunoblots were examined with chemiluminescence substrate (Western Bright Sirius; Advansta; Menlo Park, CA, United States). β-Actin (Cell signaling, Beverly, MA, United States) was used as a loading control. Finally, ImageJ software (Java-based image processing program) was utilized for the quantification of protein.

### Wound Healing Assay

A wound healing assay was carried out to estimate the migration ability of selected malignant cells using the method reported by Ko et al. (2012). For this, selected human breast cancer cells were loaded in a six-well plate followed by incubation at room temperature. The confluent malignant cells were scraped through sterile tip as well as washed through PBS and subsequently treated with 1 ml in complete medium. However, wounds were detected by bright field microscopy after 12 h incubation in obtained mixture with extracts of *R. dentatus* and many images were captured at areas flanking the intersections of the wound. ImageJ software was used to measure the gap distance of the wound at three different points and normalization of the data obtained was achieved according to the average of the control. The resultant results were finally analyzed by plotting graphs against the percentage of migration distance moved before and after treatment, normalized to control.

### Invasion Assay

Cell invasion assay was carried out with Bio-Coat Matrigel invasion assay system (BD, Bioscience) as recommended in user’s manual instructions. For this, MDA-MB-231 cells (2 × 10^5^ cells/ml) were suspended in DMEM media (serum free) and subsequently plated into the Matrigel transwell chambers consisting of polycarbonate membrane with 8 μm pores. Hereafter, the pre-incubation of cancer cells with or without plant extracts in transwell chambers was conducted for 12 h and then confined chambers were placed specifically into a 24-well plate containing different basal medium. Finally, the cells from upper wells of the transwell chambers were obliterated with autoclaved cotton swab but the cells that shifted to the lower side of the chambers were fixed followed by staining with crystal violet. Invasion ability of the malignant cells was determined by counting the cells in three randomly selected microscopic fields (magnified ×200).

### Statistical Analysis

The data were summarized as mean ± standard error of the mean (SEM) and the significant differences were analyzed using two tailed Student’s *t*-test.

## Results

### Inhibition of the Proliferation of MDA-MB-231 Cell Line by *R. dentatus*

**FIGURE 1:**
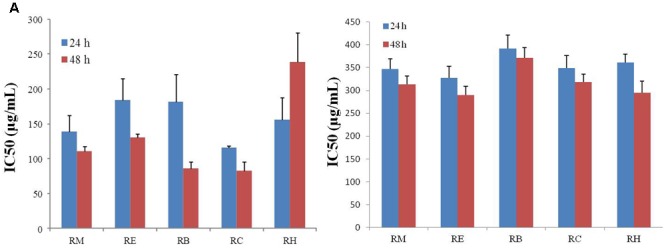
Anti-proliferative effect of five different extracts of *R. dentatus* in **(A)** MDA-MB-231 cells and **(B)** MCF-10A cells. Cancer and normal cells were seeded in 96-well plates followed by 24 and 48 h treatment with different doses of *R. dentatus* extracts. MTT assay was performed for cell viability detection. All data were expressed as mean (*n* = 3) ± SEM of three independent experiments.

The inhibition of apoptosis and tumor cell proliferation has been considered as suitable markers for the evaluation of p anti-cancer activity (Mo and Elson, 1999). For this study, *R. dentatus* was extracted with five solvents of different polarities: methanol (RM), ethanol (RE), benzene (RB), chloroform (RC), and *n*-hexane (RH). All extracts were screened for anti-proliferative activity against MDA-MB-231 and MCF-10A cells, using MTT reduction assay. Our results revealed that all the extracts suppressed the proliferation of MDA-MB-231 breast cancer cell line in a time- and concentration-dependent manner, except RH, which caused increase in cell death rate, but only with increase in time. Relative viability of breast cancer cells decreased significantly after adding different concentrations of *R. dentatus* at the same time. All *R. dentatus* extracts induced apoptosis in the cancer cells. According to the MTT assay results based on IC_50_ values, the lowest concentration of extracts that was responsible for 50% loss of cell viability in MDA-MB-231 cell line was about 116 μg/ml for the non-polar RC extract, followed by RM extract with IC_50_ of 139 μg/ml after 24 h treatment (**Figure 1A**). With 48 h treatment, RM extract had the lowest IC_50_ of 111 μg/ml, while RC extract had IC_50_ of 83 μg/ml. However, all the extracts were not cytotoxic to normal breast cells (MCF-10A) even at higher concentrations compared to those effective against MDA-MB-231 cells (**Figure 1B**). Based on these results, RM and RC extracts were chosen for further investigation in subsequent experiments.

### RM and RC Caused G0/G1 Phase Arrest in MDA-MB-231 Cells

**FIGURE 2:**
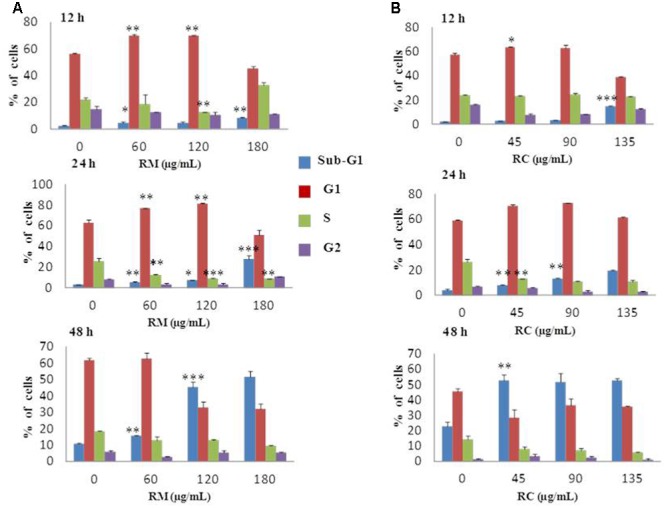
Effects of RM and RC on MDA-MB-231 breast cancer cell line after treatment for 12, 24, and 48 h. Flow cytometric analysis of **(A)** MDA-MB-231 cells with various doses of RM **(B)** MDA-MB-231 cells with various doses of RC in different phases of the cell. Cells were treated with extracts, fixed, stained with PI, and analyzed by flow cytometric analysis. All values were expressed as mean (*n* = 3) ± SEM of three independent experiments. Student’s *t*-test was used for evaluation of statistical significance (^∗^*p* < 0.05; ^∗∗^*p* < 0.01; ^∗∗∗^*p* < 0.001).

Flow cytometric analysis was conducted at 12, 24, and 48 h treatment with RM and RC extracts to ascertain whether or not the anti-proliferative effect was due to cell cycle arrest. As the effective concentrations for cell proliferation inhibition were 111 μg/ml for RM and 83 μg/ml for RC with 48 h treatment, further MTT assays were performed with three concentrations as follows: RM – 60, 120, and 180 μg/ml; RC – 45, 90, and 135 μg/ml. The studies showed that RM and RC induced dose- and time-dependent cell growth arrest and apoptosis in MDA-MB-231 cells. RM extracts produced almost similar results as RC. However, significant changes were induced in cell cycle (G0/G1 phase) after exposure with RM 60 and 120 μg/ml, relative to the control at 12 and 24 h (**Figure 2A**). Interestingly, the accumulation of cells started in sub-G1 phase after 48 h exposure with all concentrations. DNA accumulation was observed in G0/G1 phase with exposure to RC extracts (45 and 90 μg/ml) at 12 and 24 h with significant decrease of cell population in S phase (**Figure 2B**). A higher concentration (135 μg/ml) induced apoptosis at 12, 24, and 48 h. All the concentrations of RC resulted in apoptosis, as shown by a decreased proportion of cells in G1 and S phase while aggregation of cells was also observed in sub-G1 phase after 48 h treatment. On the other hand, treatment with both extracts for 24 h produced cell cycle arrest at the G1 phase.

### RM- and RC-Induced Apoptosis in MDA-MB-231 Cells

**FIGURE 3:**
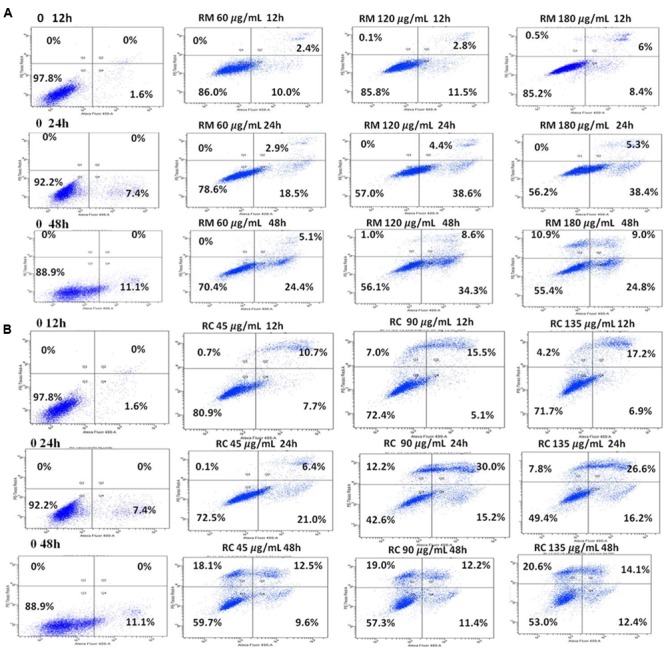
RM and RC induced apoptosis in MDA-MB-231 breast cancer cells. **(A)** RM cells and **(B)** RC cells, after treatment for 12, 24, and 48 h followed by PI and V-FITC staining. Data presented as means (*n* = 3) ± SEM of two independent experiments.

**FIGURE 4:**
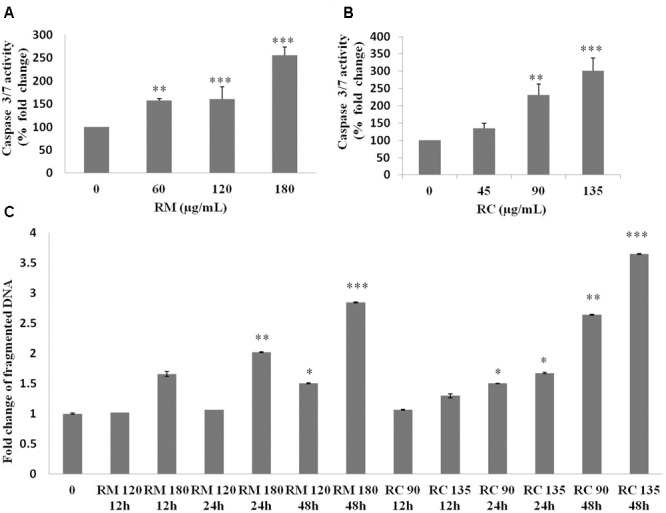
Caspase 3/7 activity and DNA fragmentation. **(A,B)** Treatment of MDA-MB-231 cells with various doses of RM and RC extracts for 48 h was carried out and caspase 3/7 activity was assessed. A significant dose-dependent increase in caspase 3/7 activity levels was observed, demonstrating the ability of extracts to induce apoptosis. **(C)** Treatment of MDA-MB-231 cells with two higher doses of RM and RC extracts for 12, 24, and 48 h was performed. Student’s *t*-test was used for evaluation of statistical variations. *n* = 3 (^∗^*p* < 0.05; ^∗∗^*p* < 0.01; ^∗∗∗^*p* < 0.001).

The ability of RM and RC to induce apoptosis was assessed by flow cytometry analysis of MDA-MB-231 cells treated with FITC-Annexin V and PI. The population of cells in each quadrant (**Figures 3A,B**) represents: necrosis (Q1), late apoptosis (Q2), live cells (Q3), and early apoptosis (Q4). It was noticed that RM and RC caused apoptosis in malignant cells depending on the time and concentration (**Figures 3A,B**). It was revealed by the kinetics of interaction with Annexin V that at 12 h treatment with RM, the proportion of early apoptotic cells (Annexin V positive cells) was considerably raised in comparison to the control cells. We observed that increase in doses (60, 120, and 180 μg/ml) increased the percentage of early apoptotic cells, likewise increase in exposure time (12, 24, and 48 h) also resulted in increased proportion of apoptosis (**Figure 3A**). However, the apoptosis-inducing effect manifested by RC was less evident in control cells compared to RM-treated cells. Interestingly, there was a discrepancy in the effect induced by RC extract in breast cancer cells. The highest % of early apoptosis was observed at 24 h treatment with all the tested concentrations; beyond 24 h, cells shifted to a late apoptotic and necrotic sub-population with RC treatment (**Figure 3B**). We further noted that when MDA-MB-231 cells were exposed for 48 h with RM and RC extracts, a significant dose-dependent increase in caspase 3/7 activity levels was observed, demonstrating its ability to induce substantial apoptosis (**Figures 4A,B**).

According to Kerr and Harmon (1991) DNA breakup is an important feature observed in cells dying apoptotically under the effect of many anticancer agents. RM (120 and 180 μg/ml) and RC (90 and 135 μg/ml)-treated cells were also assessed for DNA breakup, using the Cell Death Detection ELISA PLUS Kit. A dose- and time-dependent increase in the percentage of DNA breakup (**Figure 4C**) was observed. RC at 48 h considerably amplified the DNA breakup level by 3.6-fold, compared with the control.

### RM and RC Activate Caspase-3

**FIGURE 5:**
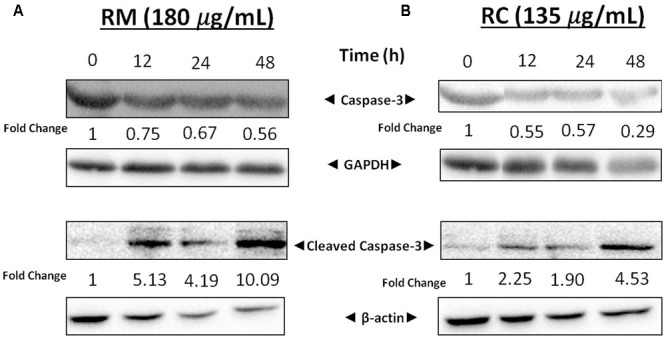
Effects of RM and RC on expression levels of cleaved caspase-3 and caspase-3. **(A)** RM (180 μg/ml) and **(B)** RC (135 μg/ml) were used to treat breast cancer cells for 12, 24, and 48 h. Results were determined through Western blot analysis. Equal loading of proteins was confirmed by normalizing with β-actin and GAPDH. The protein expression data are representative of two independent trials.

The expression levels of cellular proteins involved in apoptosis were determined in the cells treated with this extract. Western blot analysis depicted a decrease in the expression of caspase-3 associated with an increased level of cleaved caspase-3 (**Figures 5A,B**). These results further confirm that RM and RC extracts induced cell death. Caspase-3 is a key executioner of apoptosis and is one of the enzymes known for the proteolytic activation of different proteins that lead to programmed cell death (Kuribayashi et al., 2006).

### RM and RC Abrogated Constitutive *IkBa* Phosphorylation in MDA-MB-231 Cells

**FIGURE 6:**
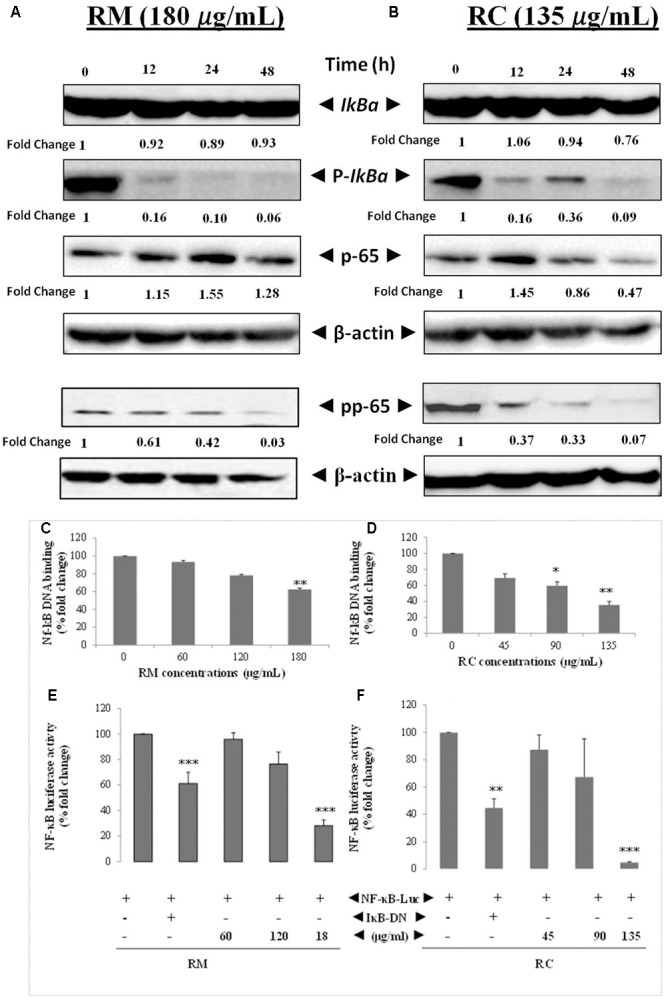
RM and RC suppressed the activation of constitutive and inducible NF-κB in MDA-MB-231 cells. **(A,B)** RM and RC inhibit the constitutive p-65 phosphorylation and activation in a time- and dose-dependent manner. MDA-MB-231 cells (2 × 10^5^ cells/ml) were treated with the indicated concentrations of RM (180 μg/ml) and RC (135 μg/ml) for 12, 24, and 48 h. Further, nuclear extracts were characterized by Western blotting in which total p-65 and phospho-specific p-65 antibodies were used. RM and RC suppressed phosphorylation and degradation of the constitutive IκBα. The indicated dose of RM and RC extracts was applied in MDA-MB-231 cells (2 × 10^5^ cells/ml) for 12, 24, and 48 h. Moreover, cytoplasmic extracts were analyzed by Western blotting in which total IκBα and phospho-specific IκBα antibodies were used. β-Actin was detected to confirm equal loading. **(C,D)** RM and RC extracts suppressed NF-κB DNA binding ability in MDA-MB-231 cancer cells. The MDA-MB-231 cells were treated with the indicated doses of RM and RC and nuclear extracts were blended and assayed for the ELISA-based DNA-binding assay. **(E,F)** NF-κB responsive elements in MDA-MB-231 cells linked to reporter gene (luciferase) were transfected with dominant-negative or wild-type IκB and exposed to the indicated doses for 24 h and luciferase activity was recorded. Luciferase trials were carried out twice, in triplicates (*n* = 3) (^∗^*p* < 0.05; ^∗∗^*p* < 0.01; ^∗∗∗^*p* < 0.001).

The effects of RM and RC on the activation of constitutive NF-κB in MDA-MB-231 cells were assessed. NF-κB is a major protein that regulates the expression of several anti-apoptotic proteins (Sethi and Tergaonkar, 2009). To determine the inhibition of *IkBa* phosphorylation and degradation, cells were treated with plant extracts and analyzed through Western blot. We found that degradation and phosphorylation of the constitutive *IkBa* were suppressed by RM and RC in a time-dependent manner (**Figure 6A**).

### RM and RC Inhibited Phosphorylation/Translocation of p-65

Phosphorylation of p-65 is required for transcriptional activity (Sethi and Tergaonkar, 2009). P-65 subunit moves to the nucleus after phosphorylation. Western blot analysis also revealed the suppression of activation induced by RM and RC in a time-dependent manner (**Figure 6B**).

### RM and RC Abrogate Constitutive NF-κB Activation in MDA-MB-231 Cells

The potential effect of RM and RC extracts on constitutively activated NF-κB in MDA-MB-231 cells was investigated through DNA binding assay. It was observed that treatment with various concentrations of RM (60, 120, and 180 μg/ml) and RC (45, 90, and 135 μg/ml) down-regulated the activity of constitutive NF-κB in a concentration-dependent manner (**Figures 6C,D**). RC 135 μg/ml reduced the NF-κB DNA binding ability by nearly 50% (*p* < 0.005). These results showed that RM and RC can alter the NF-κB activation in malignant cells. Although RM and RC reduced the activation of NF-κB by DNA binding, it is not always correlated with gene transcription regulated by NF-κB, pointing to the fact that additional regulatory mechanisms are involved in controlling NF-κB activation. To characterize the effects of RM and RC on constitutive NF-κB dependent reporter gene expression in cancerous cells, cells were transfected as described under section “Materials and Methods.” Results indicated that in the presence of RM and RC, NF-κB-dependent luciferase expression was inhibited in a dose-dependent way with highest inhibition of nearly 5% recorded at higher concentrations (**Figures 6E,F**). These results suggest that RM and RC can also abrogate reporter gene expression regulated by constitutive NF-κB.

### RM and RC Inhibited Phosphorylation of IKK in MDA-MB-231 Cells

**FIGURE 7:**
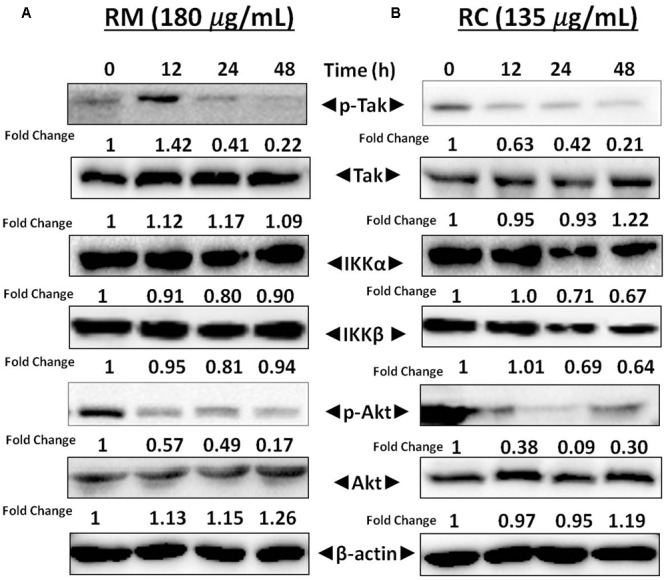
RM and RC repressed phosphorylation of Akt and TAK1 in MDA-MB-231 cells. **(A,B)** Cells were treated with RM and RC at the indicated concentration for 12, 24, and 48 h. Total lysates were analyzed by Western blot. IKKα and IKKβ proteins level were also measured. The equal loading of protein was confirmed by using β-actin as a loading control.

Activation of TAK1 leads to phosphorylation of IKKα and IKKβ (Sethi et al., 2006). Western blot analysis was performed to understand the inhibition of NF-κB in MDA-MB-231 cells by RM and RC resulting from inhibition of upstream kinase TAK1. The results showed that RMand RC suppressed phospho-TAK1 without affecting the levels of IKK proteins (**Figures 7A,B**). These results suggest that RM and RC can alter the constitutive activation of NF-κB by suppressing the upstream TAK1.

### RM and RC Suppressed Akt Activation

Akt activation is a key factor in cell survival (Yamaguchi and Wang, 2001). Therefore, it was decided to determine the effect of *R. dentatus* extracts on Akt activation in MDA-MB-231 cells. Western blot analysis depicted that RM and RC inhibited the phosphorylation of Akt, thereby suggesting that reduction in Akt activation might be a factor in apoptosis of MDA-MB-231 cells (**Figures 7A,B**). This result provides further support that *R. dentatus* can cause cell cycle arrest and cell death, since suppression of Akt activation leads to anti-survival and anti-proliferative effects.

### RM and RC Repressed the NF-κB-Regulated Cell Survival Proteins in MDA-MB-231 Cells

**FIGURE 8:**
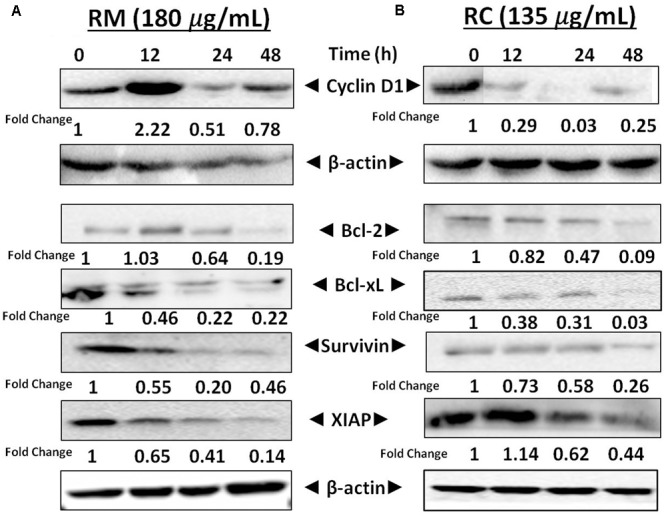
Effects of RM and RC on the regulation of NF-κB gene in MDA-MB-231 cells. **(A,B)** Cells were treated for 12, 24, and 48 h with the indicated concentrations of RM and RC extracts and Western blotting was performed to detect the proteins with specific antibodies for XIAP, survivin, Bcl-2, Cyclin D1, and Bcl-xL. The equal loading of protein was confirmed by using β-actin as a loading control. The analyzed data were obtained in triplicate (*n* = 3).

NF-κB is a well-known multi-functional protein that plays a potent role in the regulation of various proteins that are involved in cell survival (Bcl-xL, survivin, XIAP, and Bcl-2) as well as in cell proliferation [17]. The regulatory effects of NF-κB in MDA-MB-231 cells by RM and RC were investigated by Western blot analysis. It has been found that treatment with RM and RC significantly suppressed the expression of anti-apoptotic proteins, including survivin, Bcl-2, XIAP, as well as cell cycle regulator (Cyclin D1) that is clearly linked with NF-κB regulation (**Figures 8A,B**). Overall, suppressed expression was elevated in a time-dependent manner as highest down-regulation was recorded after 48 h. The treatment of MDA-MB-231 cells with RM (60 and 120 μg/ml) and RC (45 and 90 μg/ml) for 12 and 24 h resulted in slightly higher accretion of cells in the G0/G1 phase of the cell cycle. The phenomenon of apoptosis as well as cell cycle arrest in G0/G1 phase suggested reduction in cell proliferation in accord with the MTT assay results. Interestingly, down-regulation of Bcl-xL, survivin, XIAP, and Bcl-2 expression is possibly linked with the capability of RM and RC to induce apoptosis in MDA-MB-231 cells.

**FIGURE 9:**
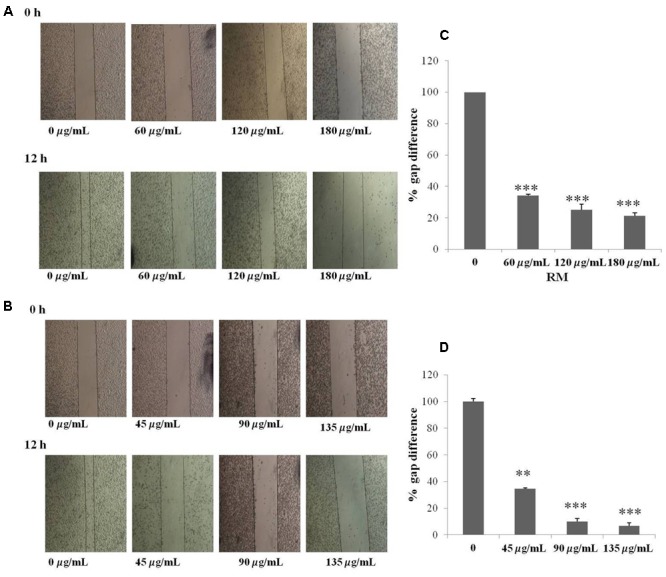
RM and RC suppressed migration of MDA-MB-231 cells. **(A,B)** The inhibitory effects of RM and RC on cell migration were evaluated using wound healing assay. Confluent monolayers of MDA-MB-231 cells were scarred and repair was visualized by microscopy after 12 h pre-treatment with the indicated concentrations of RM and RC. Thickness of wound was calculated at time zero and after 12 h of incubation, with and without RM and RC treatment. The photographs (representative) show the same areas at time zero and after 12 h of incubation. **(C,D)** Values are expressed as mean (*n* = 3) ± SEM of two independent experiments (^∗^*p* < 0.05; ^∗∗^*p* < 0.05; ^∗∗∗^*p* < 0.001).

**FIGURE 10:**
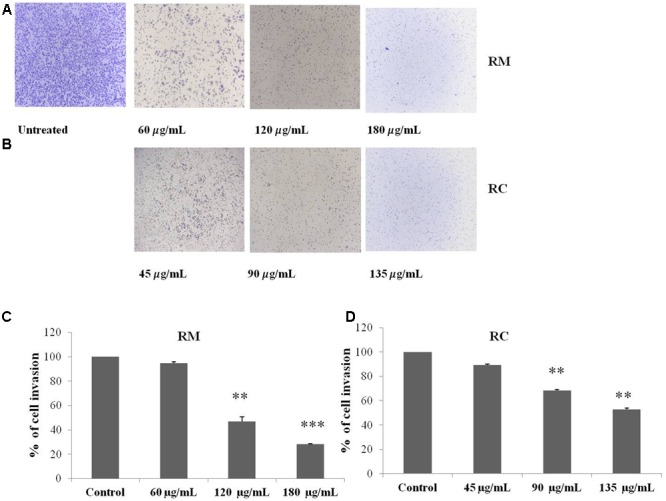
RM and RC extracts suppressed invasion of MDA-MB-231 cells. **(A,B)** Cells were seeded in the upper chamber of Matrigel. After pre-incubation with or without RM (60, 120, and 180 μg/ml) and RC extracts (45, 90, and 135 μg/ml) for 12 h, transwell chambers supplemented with 10% FBS basal medium were then positioned into the wells of a 24-well plate for 24 h and assessed for cell division. **(C,D)** Results (representative) are shown from two independent trials (*n* = 3). Each value is represented as mean percentage of invaded cells ± SEM (^∗^*p* < 0.05; ^∗∗^*p* < 0.05; ^∗∗∗^*p* < 0.001).

The regulatory role of NF-κB is well documented not only for cell proliferation but also cell invasion and migration (Helbig et al., 2003). In the present study, cell migration was investigated in MDA-MB-231 cells treated for 12 h with RM and RC. The treatment of malignant cells with both extracts resulted in considerable decrease in cell migration in a concentration-dependent manner (**Figures 9A–D**). Likewise, treatment with RM and RC extracts showed significant suppression of cell invasion (**Figures 10A,B**).

## Discussion

Natural plant products world-wide have been used as a rich source for new or novel drugs against various types of cancer (Nawab et al., 2011). Different chemotherapeutic agents with properties like induction of apoptosis and anti-angiogenesis have been isolated from natural products, e.g., epicatechin gallate from tea, curcumin from *Curcuma longa*, paclitaxel from Pacific yew, honokiol (a biphenyl extract) from *Magnolia obovata*, and emodin from *Rheum palmatum* (Li et al., 2008). Other bioactive compounds isolated from various plants have been reported to demonstrate several mechanisms of anti-cancer action, such as disturbance of multiple cellular signaling pathways, induction of apoptosis by interference with death signals, interference with metastasis and cellular invasion, as well as regulation of cell cycle (Sarkar et al., 2009; Xavier et al., 2009; Yin et al., 2013).

The present study was designed to evaluate the ability of *R. dentatus* extracts to inhibit proliferation of breast cancer cells. *In vitro* cytotoxicity tests on breast cancer cells were performed on *R. dentatus* extracted by solvents of different polarities, as reported earlier (Safarzadeh et al., 2014). The more polar extract, RM, and less polar extract, RC, were found to have the most anti-proliferative activity (as determined from the MTT assay) and were further evaluated for their cytotoxic potential.

Results indicated that RM and RC extracts induced cell cycle arrest at G0/G1 phase after 24 h at lower concentrations and caused accumulation of cells in the sub-G1 phase after 48 h. Furthermore, these extracts induced concentration- and time-dependent apoptosis in breast cancer cells as determined by flow cytometry. Apoptosis is a programmed cell death that maintains cellular homeostasis between cell death and cell division (Vermeulen et al., 2005). This physiological process prompts cellular self-obliteration, creating different morphological and biochemical features in the nucleus and cytoplasm. Additionally, treatment of MDA-MB-231 cells with RM and RC resulted in caspase-3 activation. Caspase-3 protein is responsible for cleavage of various substrates to produce morphological changes and DNA fragmentation. Observed increase in cleaved caspase-3 activation is indicative that extracts induced the intrinsic pathway of apoptosis (Goldar et al., 2015). However, regulatory mechanisms by which these extracts activate caspase-3 are still unidentified.

Activation of NF-κB is believed to be responsible for regulation of various gene products, including those involved in proliferation (Cyclin D1) and survival (Bcl-2, Bcl-xL, survivin, XIAP) (Sethi and Tergaonkar, 2009). Bcl-2 and Bcl-xL proteins were investigated as a cell death blocker that can be induced by certain chemotherapeutic drugs, thus leading to chemo-resistance (Bargou et al., 1997). The down-regulation of Cyclin D1 expression is linked with suppression of cell proliferation as well as aggregation of cells in the sub-G1 phase of the cell cycle as observed in MDA-MB-231 cells treated with RM and RC extracts. The results at the molecular level showed that RM and RC suppressed the expression of survivin, Bcl-2, Cyclin D1, and Bcl-xL in MDA-MB-231 cells. Based on the above results, we propose that *R. dentatus* may induce apoptosis by inhibition of NF-kB activation that can significantly down-regulate proliferation and anti-apoptotic proteins in MDA-MB-231 cells.

Several lines of evidence suggest that NF-κB is important in breast cancer development. NF-κB activation is also reported to inhibit apoptosis in mouse mammary epithelia (Clarkson et al., 2000). In addition, selective activation of NF-κB subunits in human breast cancer cell lines has been also reported (Cogswell et al., 2000). Moreover, it has also been shown that NF-κB inhibition in breast cancer cells can induce abrupt apoptosis (Sovak et al., 1999). Similarly, past studies reported that breast cancer tissue that didn’t show any response to chemotherapy expresses active NF-κB (Buchholz et al., 2005). The regulatory mechanism of NF-κB is linked to interaction of IκB in which inactive form of NF-κB is being sequestered in the cytoplasm. Degradation and ubiquitination of IκB occur as a result of phosphorylation of IκB by IκB kinase (IKK) thus releasing NF-κB, which then translocates to the nucleus. After translocation, it binds to several genes within the specific B-binding sites in the promoter region (Kitada et al., 1998). Our results showed that treatment of MDA-MB-231 cells with RC and RM resulted in inhibition of NF-κB activation by suppression of IKKα and IKKβ phosphorylation as well as phosphorylation and degradation of IκBα protein. Moreover, suppression of Akt by treatment with RM and RC was also observed. It has been proposed that IKK and Akt regulate the phosphorylation of p-65 (Gupta et al., 2004) and thus may be involved in the inhibitory effect of *R*. *dentatus* extracts on p-65 phosphorylation. Our study suggests that the effects of *R. dentatus* extracts on NF-κB/p-65 are through inhibition of phosphorylation and subsequent proteolysis of IκBα. Likewise, NF-κB activation also induces the expression of different molecules such as cyclooxygenase as well as certain adhesion molecules including endothelial-leukocyte adhesion molecule 1 that has been associated with malignant cell migration and invasion (Sethi and Tergaonkar, 2009). The inhibitory role of RM and RC on transcriptional activation of NF-κB and DNA binding ability in this study suggests that *R*. *dentatus* can be a potent alternative or supplement to inhibit the expression of breast cancer cells as well as other NF-κB-regulated molecules. Thus, inhibition of NF-κB activation by *R. dentatus* may account for its antiproliferative, pro-apoptotic, and chemosensitizing properties as described here. Moreover, suppression of invasion and migration potential of breast cancer cells was also observed by treatment with RM and RC.

In summary, our results clearly indicate that the chloroform and methanol extracts of *R. dentatus* can inhibit proliferation of MDA-MB-231 cells and induce apoptosis by inhibition of NF-κB activation in MDA-MB-231 cells; further detailed research will be required to isolate and identify the bioactive compounds from these extracts.

## Conclusion

Our results confirmed that *R. dentatus* extracts are able to inhibit proliferation of MDA-MB-231 cells. Overall, the most significant effects were produced by chloroform and methanol extracts of the plant. The induction of cell death by these extracts was linked with alterations in the cell cycle profile as well as by stimulation of cellular apoptosis. These potent changes were found to be related with alterations in the concentration of different antibodies, such as Cyclin D1, caspase-3, Bcl-xL, and Bcl-2. Additionally, we found that the effects of *R. dentatus* extracts on NF-κB are regulated through suppression of phosphorylation of IκBα. Our findings suggest that *R. dentatus* might play a promising role in breast cancer prevention and/or treatment. Further studies are warranted to isolate and identify potential anti-cancer compounds from *R. dentatus.*

## Author Contributions

RB and BT conceived and designed the experiment. RB performed experimental work. EA and TM helped in plant collection and interpretation of data. RB wrote the manuscript. EA helped to review the figures. BT revised the manuscript and supervised research work.

## Conflict of Interest Statement

The authors declare that the research was conducted in the absence of any commercial or financial relationships that could be construed as a potential conflict of interest.

## Abbreviations

NF-κκB: nuclear factor kappaB
RB: *Rumex dentatus* benzene
RC: *Rumex dentatus* chloroform
RE: *Rumex dentatus* ethanol
RH: *Rumex dentatus* n-hexane
RM: *Rumex dentatus* methanol
